# Sensory Lexicons and Formation Pathways of Off-Aromas in Dairy Ingredients: A Review

**DOI:** 10.3390/molecules25030569

**Published:** 2020-01-28

**Authors:** Xueqian Su, Monica Tortorice, Samuel Ryo, Xiang Li, Kim Waterman, Andrea Hagen, Yun Yin

**Affiliations:** 1 Department of Food Science and Technology, Virginia Polytechnic Institute and State University, 1230 Washington Street SW, Blacksburg, VA 24061, USA; xueqians@vt.edu (X.S.); kwater@vt.edu (K.W.); andreah2@vt.edu (A.H.); 2 Abbott Laboratories, 3300 Stelzer Rd, Columbus, OH 43219, USA; monica.tortorice@abbott.com (M.T.); samuelryo@gmail.com (S.R.); 3 Abbott Nutritional Research and Development Center, 20 Biopolis Way, Singapore 138668, Singapore; xiang.li@abbott.com

**Keywords:** off-aroma, lexicons, chemical structure, dairy, sensory, formation pathway

## Abstract

Consumers are becoming increasingly aware of the health benefits of dairy ingredients. However, products fortified with dairy proteins are experiencing considerable aroma challenges. Practices to improve the flavor quality of dairy proteins require a comprehensive understanding of the nature and origins of off-aroma. Unfortunately, existing information from the literature is fragmentary. This review presents sensory lexicons and chemical structures of off-aromas from major dairy ingredients, and it explores their possible precursors and formation mechanisms. It was found that similar chemical structures often contributed to similar off-aroma descriptors. Lipid degradation and Maillard reaction are two primary pathways that commonly cause aroma dissatisfaction. Traditional and novel flavor chemistry tools are usually adopted for off-aroma measurements in dairy ingredients. Strategies for improving aroma quality in dairy derived products include carefully selecting starting materials for formulations, and actively monitoring and optimizing processing and storage conditions.

## 1. Introduction

Flavor is one of the most important characteristics that directly determines the acceptance of foods. As the first sensory impression, a good flavor profile is critically important when consumers are first exposed to a product. The ultimate goal of any food development is to achieve a desirable flavor quality that consumers will enjoy. A good flavor profile is generally defined by an immediate sensation of an identifiable aroma, a rapid development of a balanced flavor, compatible mouthfeel and texture, and most importantly, minimal off-flavor presence [1]. 

Essentially, food flavor defects include taints and off-flavors. Taints are usually considered to be unpleasant odors and flavors imparted to food through external sources, such as the environment, packaging aids, and air. Off-flavors refer to undesirable odors and flavors imparted to food through internal deterioration. Examples include oxidation and microbial deterioration caused by improper handling and storage [2]. This review will primarily focus on the off-flavors that arise through the presence of aroma substances, that is, the off-aromas. Off-aromas usually occur when the concentration of a volatile compound in the food matrix is so high that it exceeds the general tolerance of human subjects. This off-note concentration at which consumers begin to reject the product is called the rejection threshold. Generally speaking, the rejection threshold more sensitively reflects the consumers’ preference towards one product when compared to the detection threshold and the recognition threshold [3,4]. Off-aromas create severe issues and affect food product quality along the supply chain. Off-aroma presence has also been reported to be the most common reason for consumer rejection of products, and it accounts for the largest percentage of complaints in the food industry [5]. In order to avoid financial loss to manufacturers and improve consumer confidence in brand images, minimizing unpleasant flavor experiences in food products becomes a primary mission in the modern food industry. 

Many ingredients fall into the dairy category, including both dried and fluid dairy proteins. Whey and milk proteins, caseins, and serum proteins are well-known dried dairy ingredients. Fluid ingredients, such as whole milk, low-fat milk, and non-fat milk, are also included in the dairy category [6]. Although there has been increasing consumer interest in plant-based proteins due partially to changing consumer behavior and concerns about health and animal welfare, the dairy protein industry is still growing and generating great economic value [7,8]. According to the Global Dairy Protein Market 2019 Industry Research Report, the global dairy protein market was worth $15.04 billion in 2017 and is estimated to be valued at $19.88 billion by 2023. This represents a 5.8% increase in the annual compound growth rate. The dairy protein market can be segmented into various sectors based on ingredients and applications, and the food and beverage branch is constantly expanding and innovating [9]. Also, dairy ingredients are being utilized to develop a large portion of high-protein drinks. While the nutritional value is highly appreciated, products fortified with dairy proteins face significant challenges in flavor satisfaction. In fact, the addition of protein to a food product may impart undesirable off-aromas or change its original aroma profile due to interaction, binding or the release of volatiles [10]. Flavoring high-protein foods has been particularly challenging to food industry because of the various aroma origins, from both raw materials and from processing and storage. Aroma imbalance and fading are commonly observed issues. 

Taking proactive approaches throughout the product development stage will encourage off-aroma prevention. Senior flavor scientists have advised food manufacturers to partner with flavor suppliers to develop solutions early in the process. More importantly, off-aroma knowledge should be disseminated to a wider group of audiences, including ingredient suppliers, product developers, processing engineers, sensory scientists, marketing professionals, and consumers, in order to overcome the aroma dissatisfaction associated with dairy ingredients. Sensory lexicons play an important role in knowledge dissemination among diverse audiences. Building bridges between off-aroma lexicons, chemical natures, and formation mechanisms will not only assist effective communication between different parties but will also aid the targeted resolution of aroma imperfection and improve the sensory quality for final products. Furthermore, understanding the precursors and formation mechanisms will provide opportunities for accurately locating off-aroma occurrence throughout the supply chain and enable immediate problem-solving actions. Several review articles provide insightful discussion on aroma and sensory challenges in dairy-related products [1,6,11,12]. However, to our best knowledge, a comprehensive picture of chemistry and sensory descriptors for off-aroma compounds is not yet available. This review will fill in the abovementioned knowledge gap. 

## 2. Investigating Off-Aroma Sensory Descriptions and Their Chemical Natures in Dairy Proteins 

The primary quest in flavor research is to characterize chemical compounds that provide specific sensory attributes to the aroma of foods [10]. Relating the chemical and sensory responses of off-aroma in foods is one of our major focuses. Sensory perception is heavily influenced by cultural and emotional experiences. The actual sensation process is largely determined by the physicochemical nature of volatile compounds responsible for off-notes, their concentration in food matrices, and the sensitivity of human subjects [13]. Besides traditional sensory panels, off-note lexicons can also be generated from an olfactometer equipped to a gas chromatograph (GCO). The odors of effluent volatile compounds from the separation column are detected and evaluated by the human nose, thereby establishing an association between the lexicons and the chemical structures [14,15]. The positive identification of the chemical structures of unknown volatiles occurs by matching the mass spectrum, odor attributes, and retention indices against authentic aroma standards. GCO has been used as an effective tool for unveiling the chemical structures and odors of off-note volatile compounds in dairy ingredients. For example, “cheesy”, “potato”, “popcorn”, and “cabbage” were perceived as major off-aroma descriptors by use of an olfactometer. By comparing these odor attributes and mass spectrum with aroma standards, these descriptors were respectively confirmed to be butanoic acid, methional, 2-acetyl-1-pyrroline, and dimethyl trisulfide [16].

**Figure 1:**
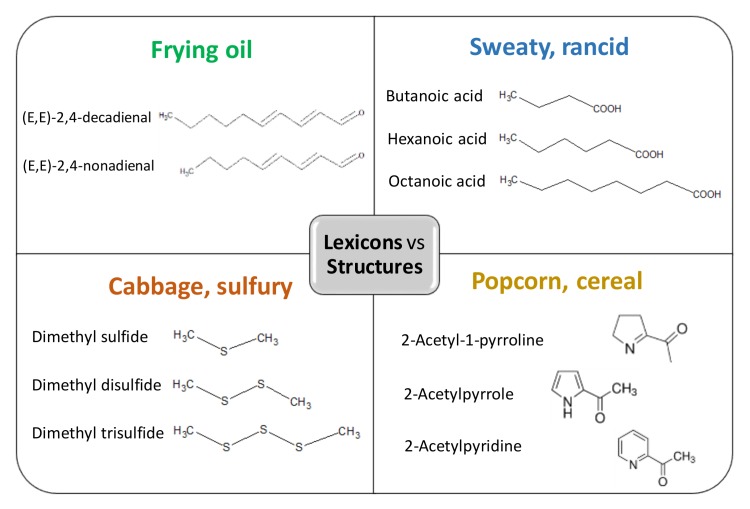
Chemical structures of off-aroma compounds grouped by sensory lexicons.

The chemical natures and sensory lexicons of selected off-note volatile compounds are listed in Table 1. They are either preexisting in raw ingredients or formed during product manufacturing or storage. Some lexicons, like “rancid”, “green”, “garlic”, and “vinegar”, are commonly found in many dairy ingredients. This is not surprising since the compositional profiles of dairy ingredients are relatively similar to their constituent components of protein, fat, and lactose. The odor attributes of some compounds are described using similar lexicons, especially those that belong to the same homologous series. For example, the odor attributes of (*E*,*E*)-2,4-decadienal and (*E*,*E*)-2,4-nonadienal were both recorded as “frying oil”, and the acids generally exhibit a “sweaty” and “rancid” note (Figure 1). It might be helpful for sensory scientists and product developers to keep in mind that structurally similar compounds are likely to exhibit similar or identical sensory characteristics. However, exceptions do occur: Hexanal (C6) and octanal (C8) display distinctly different odor qualities because their excitation mechanisms on olfactory receptors are different [17,18]. Special attention should be paid to sulfur-containing volatile compounds having “cabbage” and/or “sulfurous” odors, as they can be significant off-aroma contributors at extremely low odor thresholds. It is also worth mentioning that the discussion of off-note compounds should be based on the particular food matrix or ingredient because a compound considered as an off-aroma in one food might have a desirable note in another [19]. “Popcorn” and “cereal” odors owing to the presence of heterocyclic volatiles, including 2-acetyl-1-pyrroline, are considered to be off-aromas or foreign smells in dairy ingredients, because product developers generally prefer starting materials with a “plain” or “clean” flavor profile. However, 2-acetyl-1-pyrroline is a characteristic compound found in aromatic rice, bakery goods, and seafood. Although the complex chemistry makes off-note solutions in dairy ingredients a challenging task, demonstration of the association between lexicons and chemical structures will enhance our understanding of the nature of off-aromas and assist problem-solving strategies. 

## 3. Understanding the Mechanisms Involved in Primary Formation Pathways of Off-Aromas in Dairy Proteins

The off-aromas in dairy products primarily originate from the degradation of major milk constituents, including lipids, protein, carbohydrates, minerals, and vitamins. Elucidating the formation mechanisms of aroma defects from such a complex matrix involving many interactions is challenging. Milk, the starting material for almost all dairy ingredients, has a composition of 87% water, 5% lactose, 4% fat, 3% protein, and 1% ash. Many flavor researchers agree that the primary sources of off-aroma formation in dairy products are from lipid and protein degradation [11,25,27,30].

### 3.1. Lipid Degradation

Lipid degradation in dairy products is a major cause of deterioration not only due to its undesirable implications for human health but also because it causes decreased overall quality and consumer acceptance [31]. Lipids, present in trace amounts in dairy ingredients, can become significant precursors for off-aromas. The degree of lipid degradation is influenced by water activity, temperature, oxygen, and light [32]. The formation of off-aromas from lipids usually occurs through two routes: Autoxidation and lipolysis [31,33,34]. The formation mechanisms, precursors, and odor thresholds of selected volatile compounds generated from lipid degradation (autoxidation and lipolysis) are compiled in Table 2.

#### 3.1.1. Flavor Significance and Formation Mechanisms of Autoxidation

Autoxidation is the oxidation of unsaturated lipids. Its reaction with molecular O_2_ results in the formation of hydroperoxides, which then break down to off-aroma compounds [35]. The widely accepted pathway consists of three stages: Initiation, propagation, and termination [36]. Initiation occurs in the presence of initiators, such as heat, light, and metal. The unsaturated lipid molecules lose a hydrogen atom and produce a carbon-centered alkyl radical. The alkyl radical reacts rapidly with oxygen to form the peroxy radical, which then attacks a new lipid molecule to form hydroperoxide and propagates the chain reaction [37]. This self-propagating and self-accelerating process is repeated until no hydrogen source is available or the chain is interrupted. Hydroperoxides are produced as the primary oxidation products during propagation, and they are odorless and very unstable. The decomposition of hydroperoxides is believed to involve homolytic cleavage between oxygen and oxygen bonds. The resultant alkoxy radical undergoes β-scission on the carbon–carbon bond and forms oxo-compounds and alkyl radical. After the electron rearrangement, a wide range of secondary lipid oxidation products, including aldehydes, ketones, acids, alcohols, and furans, are produced [36,38] (Table 2). Many of these products have been reported as contributing to off-aromas in dairy products due to their low odor detection thresholds. 

Aldehydes are the most significant breakdown volatiles from alkoxy radicals. In general, their odor thresholds are relatively low (Table 2), making them potent compounds to the overall aroma profile. Often described as “green”, “metallic”, and “fatty”, they are responsible for the undesirable flavors in lipid-containing foods, including dairy products. The final structures of lipid-derived aldehydes depend on the fatty acid precursors, the formed hydroperoxide, and the stability of decomposition products [37]. Multiple generation pathways could be involved in the formation of a particular aldehyde. For instance, the autoxidation of linoleic acid generates 9- and 13-hydroperoxides. Cleavage of 13-hydroperoxide will lead to hexanal, and the breakdown of 9-hydroperoxide will lead to 2,4-decadienal [24,39]. However, the subsequent retro-aldol reaction of 2,4-decadienal will also produce hexanal [37]. Alcohols are formed via cleavage of lipid hydroperoxides during autoxidation of fatty acids [37,38,40]. Although aliphatic alcohols usually have a negligible influence on the overall off-aromas, alcohols like 1-octen-3-ol and 1-penten-3-ol were reported to be important off-aromas in dairy products [25,41]. One of the ketones that contributes significantly to off-aromas in whey and milk proteins is 1-octen-3-one [20,27], which has a “mushroom” note. Aliphatic ketones are generally formed by lipid autoxidation [37]. However, the formation mechanism for some ketones, like (*Z*)-1,5-octadien-3-one and (*E*,*E*)-3,5-octadien-2-one, are seldom reported [42]. Furans are well-known autoxidation products from linoleic acid [13,37]. Obviously, linoleic acid is an important precursor for off-note generation (Table 2). A similar perspective was reported by Jeleń (2006) [13] and Kochhar (1996) [43]. Due to the large abundance in foods and high susceptibility to oxidation, linoleic acid and its glycerides are among the most important precursors for aldehyde compounds.

#### 3.1.2. Flavor Impact and Formation Mechanisms of Lipolysis

**Figure 2:**
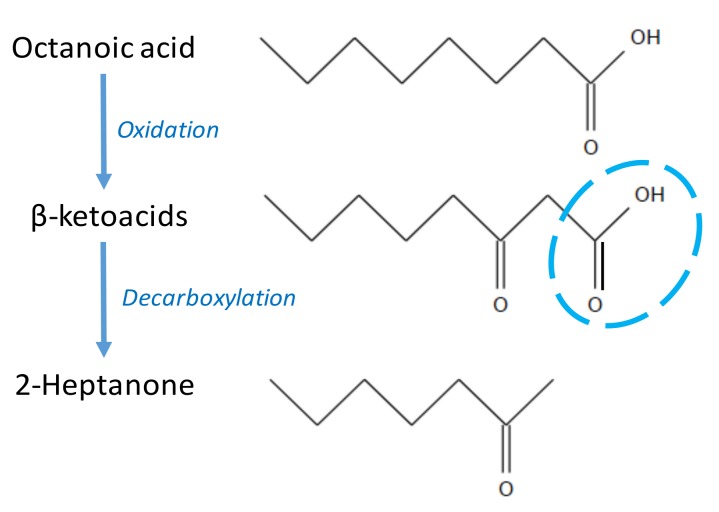
The formation of heptanone through the lipolysis pathway as an example of methyl ketone formation from β-oxidation and decarboxylation of fatty acids.

Lipolysis is the hydrolysis of triglycerides, the major lipid component of milk, and it is catalyzed by lipases [33]. The lipases that cause problems in milk and dairy products are from two main types: Lipoprotein lipase, which naturally occurs in raw milk, and bacterial lipases produced predominantly by psychrotrophic bacteria due to contamination. Milk lipase can be inactivated by pasteurization, but bacterial lipases are heat stable, meaning they can survive through processing and cause lipolysis during storage [34]. The hydrolysis of lipids in milk produces free fatty acids, partial glycerides, and possibly glycerol. The free fatty acids cause both undesirable and desirable properties: Short-chain fatty acids, such as butanoic acid and hexanoic acid, are responsible for the off-aromas known as “vinegar”, “cheesy”, “sweaty”, and “soapy” in dairy products [44]. However, in the manufacture of Parmesan and Romano cheese, lipases are used to produce fatty acids that contribute to the characteristic piquant flavor [34]. Besides short-chain fatty acids, methyl ketones are an important volatile group derived from lipolysis [33,34,45]. Their generation pathway involves fatty acid oxidation to β-ketoacids, followed by decarboxylation to corresponding methyl ketones with one carbon atom less [11,46] (Figure 2). Similar to free fatty acids, methyl ketones contribute to the characteristic aroma of blue-veined cheese [47]. However, they can have a negative influence on the flavor profile of milk products, especially Ultra-high temperature milk [48]. Both methyl ketones and acid groups have a wide range of odor detection thresholds, from ppm to ppt levels. Therefore, the contribution of the two groups to dairy aroma profiles, to a great extent, depends on the attributes of an individual compound. For example, 2-heptanone, with an odor threshold of 1.3 ppb in air, is an important aroma to the “cooked” note in UHT milk. Acetic acid, with a “vinegar-like” odor property, was found to be a significant off-aroma in whey protein concentrates and isolates [20]. Compounds, such as 2-pentanone and 2-decanone, are not likely to be significant contributors to off-aromas because of their relatively high detection thresholds.

### 3.2. Maillard Reaction

Maillard reaction is a vitally important class of chemical deterioration in dairy products. Nonenzymatic browning generally occurs during heat processing, such as pasteurization, and storage at moderate to high temperatures. The reaction requires a carbonyl group from a reducing sugar and an amino group from a protein, peptide, or amino acid. Specifically, it involves the formation of unstable glycosylamine from the condensation of carbonyl and amino groups, and the Amadori compound is formed from the rearrangement of glycosylamine. The Amadori compound then undergoes various reaction pathways, including fissions, dehydration, and condensation, before generating desirable and undesirable flavors [10,69,70]. Lactose is usually the primary reducing sugar in dairy ingredients involved in Maillard reaction [44]. Milk proteins and lactose subjected to Maillard browning generate a wide variety of odorants, namely Strecker aldehydes, sulfur- and nitrogen-containing compounds, maltol, and diacetyl [28,71]. Off-aromas generated from Maillard reaction in dairy products are shown in Table 3. Amino acids and sugars are the exclusive precursors for almost all the undesirable flavors. Amino acids, especially sulfur-containing cysteine and methionine, are primary precursors of compounds responsible for “garlic”, “cabbage”, “potato”, and “popcorn” notes. Sulfur-containing off-aromas are frequently studied due to their extremely low odor thresholds and sensory importance. Methional, methanethiol, and dimethyl sulfide are formed from Maillard reaction by Strecker degradation [72] from methionine. Methanethiol is then oxidized to dimethyl disulfide and dimethyl trisulfide progressively [23]. Interestingly, the odor thresholds of dimethyl sulfide, dimethyl disulfide, and dimethyl trisulfide decrease dramatically as oxidation progresses, suggesting that the off-aroma profile might change greatly during storage. As a result, the oxygen content in the environment might affect the sensory attributes of dairy products significantly, by impacting the oxidation rate of Maillard reaction compounds. Some aldehydes, like 3-methylbutanal, 2-methylbutanal, 2-methylpropanal, and phenylacetaldehyde, are Strecker degradation products of leucine, isoleucine, valine, and phenylalanine, respectively [73,74]. Besides, many heterocyclic compounds, such as 2-acetyl-1-pyrroline, 2-acetyl-2-thiazoline, and 2-propionyl-1-pyrroline, have low odor detection thresholds and are considered to be potent volatile compounds. 

Various parameters, including pH, time, temperature, and water activity, are known to influence the overall outcome of Maillard reaction: In general, alkaline conditions, intermediate water activity (0.5–0.8), elevated temperature, and prolonged time increase the rate of Maillard reaction [75]. The nature of the reactants also has a direct influence on the rate of Maillard browning. For instance, lysine and glycine allow for a higher degree of reaction compared to cysteine [75]. It is important to keep in mind that Maillard reaction is not only the cause of off-aromas but is also a rich source of desirable flavors [69]. In conclusion, depending on the sensory expectation of a particular food system, Maillard browning could be tailored to either promote or inhibit reactions in order to achieve a particular sensory goal for products.

## 4. Analytical Methods for Measuring Off-Aromas

### 4.1. General Methods

The detectable odor thresholds for off-note volatile compounds are usually at parts-per-million (ppm), parts-per-billion (ppb), or even parts-per-trillion (ppt) levels. Therefore, identification and determination of off-aromas becomes challenging and requires sensitive instrumentation. Due to the nature of volatiles, the regular toolbox used by flavor chemists is usually sufficient for off-aroma analysis. Based on the differences in the polarity of odorants and various physical properties of matrices, extraction of aroma or off-aroma from a wide range of samples could be achieved with many approaches, including direct solvent extraction, liquid-liquid extraction, solid-phase extraction/microextraction, and stir bar sorptive extraction. Liquid-liquid extraction is a time-consuming approach and may cause decomposition of unstable volatiles. It is occasionally used owing to its relatively low equipment investment. Solid-phase extraction/microextraction and stir bar sorptive extraction are popular techniques because of their simple sample preparation, acceptable reproducibility, and environmentally friendly nature [96]. Stir bar sorptive extraction has higher sensitivity compared to solid-phase microextraction due to the large phase ratio between the sample and stir bar coating [97]. Solvent-assisted flavor evaporation is also a good option because of its exhaustive volatile extraction and minimal thermal artifacts during isolation [98,99]. Many studies employed direct solvent extraction plus solvent-assisted flavor evaporation [20,21,23] or solid-phase microextraction [31,50,100,101] for determining off-aromas in dairy ingredients. The identification and quantitation of odorants, as previously discussed, is usually achieved through well-established flavor research instrumentation, such as gas chromatography-mass spectrometry (GC-MS), flame ionization detectors (GC-FIDs), or pulsed flame photometric detectors (GC-PFPDs) equipped with olfactometry. It is worth noting that PFPD has the capability of sensitively and selectively detecting low concentration sulfur-containing compounds [102].

### 4.2. Novel Approaches

Several novel flavor analysis methods have been developed and refined over the past few decades in order to resolve the major disadvantages of classic GC methods, such as limited separation and resolution, tedious sample pretreatment, and aroma isolation. As a high-resolution analytical method, multidimensional gas chromatography (MDGC) has evolved into promising technology to enhance the resolving power of aroma analysis by incorporating multiple separation dimensions; namely, more than one GC column [103]. Since a considerable challenge for aroma analysis is overlapping chromatographic peaks resulting from complex matrices, the MDGC technology can improve the separation of samples of interest and enhance identification more reliably. While MDGC is innovative for advanced separation and detection, adoption of this method is primarily restricted to academic settings, the industry application is still limited. Nevertheless, MDGC is a potential approach to improve the understanding of aroma perception, and it could be adapted for rapid determination of off-aromas in the dairy industry. On-line chemical ionization mass spectrometry (on-line CIMS) has become a powerful tool in real-time detection of food-related aromas. The real-time detection advantages of on-line CIMS make it feasible to explore the aroma release dynamics from food. More importantly, this highly sensitive technique enables samples to be analyzed without mandatory yet time-consuming pretreatment practices from routine aroma extraction and enrichment, so the sampling frequency of the technique achieves several hertz (Hz) [104]. However, this technique is not ideal for chemical–structural elucidation when compared to classic and specific GC-MS approaches [104]. Additionally, the food industry has started to incorporate machine olfaction as a regular instrumental operation for quality control, research, and development. The electronic nose has been successfully applied for evaluating fresh flavor in milk [105] and off-aroma in pineapples [106]. In general, machine olfaction devices are less time-consuming, more portable, and cost effective compared to traditional analytical methods and sensory panels. However, the complete replacement of human sensory perception with machine olfaction is not yet possible [107].

Advanced data analysis techniques have been incorporated in understanding and predicting aroma behaviors in foods [108,109,110]. An accurate predictive model was developed by Viry et al. (2018) [109] for flavor partitioning and protein–flavor interactions in fat-free dairy solutions. Chen, Husny, and Rabe (2018) processed raw instrumental data and examined its correlation to sensory results by use of the machine learning approach, and successfully predicted the fishiness off-flavor in dairy powders [110]. These novel data processing approaches are receiving increasing attention and may soon be widely recognized and adopted for dairy ingredients.

## 5. Strategies to Minimize Off-Notes in Dairy Ingredients

The flavor of dairy ingredients is of significance because off-aromas will be carried into the finished products [20] and become problematic for consumer acceptability. Off-aromas can be introduced at the milk origin, processing, handling, and storage stages. The effect of processing and storage treatments on off-note generation is significant. Heating temperature and time, oxygen exposure, water activity, packaging materials, and lighting conditions will all have direct or indirect influences on the reaction rate of lipid oxidation, Maillard reaction, and sugar degradation. Improving the understanding of sensory descriptors and chemical natures of off-aroma as well as investigating their formation pathways would be the fundamental approach to unveil flavor deterioration. For instance, by knowing an elevated temperature during thermal processing is a preferred condition for Maillard reaction, the manufacturers might be able to mitigate or at least reduce a “potato” off-note in a finished bottle by decreasing the processing temperature. In short, selecting ingredients with caution and optimizing manufacturing conditions with specific targets can be helpful for minimizing undesirable flavor impacts in the final dairy-related products. 

## 6. Conclusions

This review established the connection between off-aroma lexicons and chemical structures existing in dairy ingredients. Furthermore, the possible formation precursors and mechanisms of major undesirable odorants were explored and compiled. With the growth of consumer interest in dairy protein ingredients, a good understanding of off-aromas and their generation pathways is believed to be useful in overcoming the flavor challenges of high protein formulations. Many off-aromas are break-down compounds from proteins, fats, and sugars via lipid degradation and Maillard reaction pathways. In order to minimize off-aromas over time, manufacturers should carefully select the starting dairy ingredients for protein-based products and adjust the processing parameters to decrease the rate of flavor degradation. Controlling storage conditions will also be helpful for minimizing off-note development. Measuring undesirable odorants in dairy ingredients is time-consuming and detail-demanding work despite the recent development of new technologies. The majority of off-aroma investigation is still performed by use of classic flavor analysis tools. The review will facilitate solution development to effectively control off-note formation and improve consumer experiences regarding dairy-related products.

## Figures and Tables

**Table 1:** Sensory lexicons and chemical natures of selected off-aroma compounds in major dairy ingredients.

Ingredients	Major Off-Aroma Lexicons	Chemical Compounds	References
Whey protein concentrate and isolate	Cheesy/Rancid	Butanoic acid	[16]
	Popcorn	2-Acetyl-1-pyrroline	[16]
	Maple/Spicy	Sotolon	[16]
	Cucumber	(*E*,*Z*)-2,6-Nonadienal	[16]
	Cucumber/Old books	(*E*)-2-Nonenal	[16]
	Cabbage	Dimethyl trisulfide	[16,20]
	Garlic	Dimethyl trisulfide	[21]
	Vinegar	Acetic acid	[20]
	Mushroom	1-Octen-3-one	[21]
	Fatty/Smoky	2-Methoxy phenol	[21]
	Fatty	Decanal	[21]
	Fatty/Stale	Decanoic acid	[21]
	Cilantro/Waxy	γ-Nonalactone	[21]
Whey Protein Hydrolysates	Sulfur	Dimethyl sulfide	[22]
	Potato	Methional	[22]
	Burnt/Smoky	Guaiacol	[22]
Sweet whey powder	Vinegar-like	Acetic acid	[23]
	Grassy	Heptanal	[23]
	Cooked potato	2,5-Dimethylpyrazine	[23]
	Fried	2-Propionyl-1-pyrroline	[23]
Liquid cheddar whey	Green	Hexanal	[24]
	Popcorn	2-Acetyl-1-pyrroline	[24]
	Potato	Methional	[24]
	Frying oil	(*E*,*E*)-2,4-Decadienal	[24]
	Frying oil	(E,E)-2,4-Nonadienal	[24]
Serum protein concentrate	Green/Earthy	Hexanal	[21]
	Potato	Methional	[21]
	Mushroom	1-Octen-3-one	[21]
	Fatty/Smoky	2-Methoxy phenol	[21]
	Cucumbers	(*E*)-2-Nonenal	[21]
	Fatty	Decanal	[21]
	Fatty/Stale	Decanoic acid	[21]
	Cilantro/Waxy	γ-Nonalactone	[21]
Milk protein concentrate	Potato	Methional	[25]
	Popcorn	2-Acetyl-1-pyrroline	[25]
	Carpet/Clay	Benzothiazole	[25]
	Vinegar-like	Acetic acid	[25]
Milk protein isolate	Burning plastic	2-Methyl-1-propanol	[25]
	Popcorn	2-Acetyl-1-pyrroline	[25]
	Cabbage/Garlic	Dimethyl trisulfide	[25]
	Carpet/Clay	Benzothiazole	[25]
	Garbage	Propanoic acid	[25]
Nonfat dry milk	Burnt sugar	Furaneol	[26]
	Rancid	Butanoic acid	[26]
	Grape	*o*-Aminoacetophenone	[26]
	Metallic	(*E*)-4,5-Epoxy-(*E*)-2-decenal	[26]
	Sweaty	Pentanoic acid	[26]
Whole milk powder	Cheesy/Rancid	Butanoic acid	[27]
	Popcorn	2-Acetyl-1-pyrroline	[27]
	Maple/Spicy	Sotolon	[27]
	Mushroom	1-Octen-3-one	[27]
	Potato	Methional	[27]
	Sweaty	Hexanoic acid	[27]
	Sweaty	Octanoic acid	[27]
	Grape	*o*-Aminoacetophenone	[27]
	Fecal/Mothball	3-Methyl indole	[27]
UHT milk	Cooked/Malty	3-Methylbutanal	[28]
	Barny/Brothy	Furfural	[28]
	Cooked	2-Heptanone	[28]
	Earthy/Fatty	Heptanal	[28]
	Cooked/Nutty	Benzaldehyde	[28]
	Garlic/Cabbage	Dimethyl trisulfide	[28]
	Earthy/Barny	*p*-Cresol	[28]
	Grass	Octanal	[28,29]

**Table 2:** Odor thresholds and formation mechanisms of off-aroma compounds derived from lipid autoxidation and lipolysis.

Chemical Groups	Volatile Compounds	Odor Thresholds	Precursors	Formation Mechanisms
Aldehydes	Heptanal	250 ppt in air [49]	Oleic acid [50]	Autoxidation [50]
	Nonanal	4.5 ppt in air [49]	Oleic acid [50]	Autoxidation [37,50]
	Octanal	7.8 ppt in air [49]	Oleic acid [50]	Autoxidation [37,50]
	Pentanal	39 ppt in air [51]	Linoleic acid [38]	Autoxidation [38]
	Hexanal	30 ppt in air [52]	Linoleic acid [38,50]	Autoxidation [38,50]
	(*E*,*E*)-2,4-Decadienal	0.04–0.16 ppt in air [53]	Linoleic acid [38]	Autoxidation [38]
	(*E*,*Z*)-2,4-Decadienal	0.04–0.16 ppt in air [53]	Linoleic acid [38]	Autoxidation [38]
	(*E,Z*)-2,6-Nonadienal	3.8 ppb in oil [54,55]	Linolenic acid [42]	Autoxidation [42]
	Propanal	690 ppt in air [49]	Linolenic acid [11]	Autoxidation [37,38]
	(*E*)-2-Hexenal	480 ppt in air [49]	Linolenic acid [38]	Autoxidation [38]
	Benzaldehyde	350–3500 ppb in water [42]	2,4-Decadienal [13]	Autoxidation [13]
	(*Z*)-4-Heptenal	0.2 ppb in water [56]	(*E,Z*)-2,6-Nonadienal [57]	Retro-aldol condensation [57]
Alcohols	Heptanol	3 ppb in water [58]	Oleic acid [38]	Autoxidation [38]
	Hexanol	2.5 ppm in water [42]	Linoleic acid [59]	Autoxidation [59]
	Pentanol	4 ppm [42]	Linoleic acid [38]	Autoxidation [38]
	1-Penten-3-ol	4.3 ppb in air [49]	Linolenic acid [60]	Autoxidation [60]
	1-Octen-3-ol	48 ppt in air [49]	Linoleic acid [38]	Autoxidation [38]
Ketones	1-Octen-3-one	0.03–1.12 ppt in air [54]	Linoleic acid or linolenic acid [13]	Autoxidation [61]
	1-Penten-3-one	1.3 ppb in water [62]	Linolenic acid [60]	Autoxidation [60]
	(*Z*)-1,5-Octadien-3-one	0.003–0.006 ppt in air [54]	Linolenic acid [42]	NA
	(*E,E*)-3,5-Octadien-2-one	<17 ppb [63]	NA	NA
Methyl ketones	2-Heptanone	1.3 ppb in air [49]	Triglycerides [11]	Lipolysis [11,46]
	2-Pentanone	70 ppm in water [58]	Triglycerides [11]	Lipolysis [11,46]
	2-Hexanone	76 ppb in air [64]	Triglycerides [11]	Lipolysis [11,46]
	2-Octanone	50 ppb in water [42]	Triglycerides [11]	Lipolysis [11,46]
	2-Decanone	0.16-5.5 ppm [65]	Triglycerides [11]	Lipolysis [11,46]
	2-Nonanone	1.7 ppb in air [49]	Triglycerides [11]	Lipolysis [11]
**Acids**	Butanoic acid	240 ppb in water [58]	Triglycerides [11]	Lipolysis [61]
	Acetic acid	60 ppt in air [66]	Triglycerides [11]	Lipolysis [11,46]
	Hexanoic acid	3 ppm in water [67]	Triglycerides [23,68]	Lipolysis [23,68]
	3-Methylbutanoic acid	1.5 ppt in air [66]	Triglycerides [23,68]	Lipolysis [23,68]
**Furans**	2-Pentylfuran	270 ppt in air [49]	Linoleic acid [37]	Autoxidation [37]
	2-Ethyl furan	2–27 ppm [65]	2,4-Decadienal [13]	Autoxidation [13]

NA: Not available.

**Table 3:** Off-aromas generated from the Maillard reaction pathway in dairy ingredients.

Dairy Products	Off-Aromas	Odor Attributes	Odor Threshold	Precursors	Off-Aroma References
Liquid whey	Dimethyl trisulfide	Garlic	0.01 ppb in water [76]	Methionine [72]	[24]
	2-Acetyl-1-pyrroline	Popcorn	0.1 ppb in water [42]	Proline [77]	
	Methional	Potato	0.1–0.2 ppt in air [78]	Methionine [23]	
	2-Methoxy-3-isopropylpyrazine	Earthy	0.0005–0.001 ppt in air [79]	Peptides or free amino acids [80]	
Milk protein concentrate and isolate	Sotolon	Spice	0.015 ppt in air [66]	Glutamic acid and pyruvate [81]	[26]
	Benzothiazole	Carpet	80 ppb in water [42]	Sulfur-containing precursors	
	2-Aminoacetophenone	Tortilla	0.2 ppb in water [82]	Tryptophan [82]	
	3-Methylbutanal	Malty	3–6 ppt in air [79]	Leucine [73]	
	2-Methylbutanal	Cocao	1 ppb in water [83]	Isoleucine [84]	
Nonfat dry milk	2-Acetyl-2-thiazoline	Popcorn	0.016–0.022 ppt in air [79]	Cysteine or cystine [77]	[26]
	2-Acetylthiazole	Popcorn	10 ppb in water [85]	Cysteine [84]	
Whey protein concentrate	Diacetyl	Buttery	5 ppt in air [49]	Glucose and proline [86]	[21]
	Dimethyl disulfide	Garlic	0.16 ppb in water [87]	Methionine [72]	
	2-Methyl-3-furanthiol	Vitamins	0.0025 ppt in air [56]	Multiple origins [88]	
	2-Acetylpyridine	Popcorn	19 ppb in water [89]	Cysteine [84]	
	Phenylacetaldehyde	Floral	0.6–1.2 ppt in air [78]	Phenylalanine [74]	
Sweet whey powder	2,6-Dimethylpyrazine	Cooked meat	200–9000 ppb in water [90]	Peptides or free amino acids [80]	[23]
	2,5-Dimethylpyrazine	Cooked potato	0.8–1.8 ppm in water [90]	Peptides or free amino acids [80]	
	2-Ethylpyrazine	Roasted nuts	6–22 ppm in water [90]	Peptides or free amino acids [80]	
	2,3-Dimethylpyrazine	Nutty	2.5–35 ppm in water [90]	Peptides or free amino acids [80]	
	2-Propionyl-1-pyrroline	Fried	0.02 ppt in air [77]	Proline [77]	
Whey protein hydrolysates	Dimethyl sulfide	Sulfur	1.0 ppb in water [85]	Methionine [72]	[22]
	Dimethyl trisulfide	Cabbage	0.06–1.2 ppt in air [78]	Methionine [72]	
	3-Methylbutanal	Malty	3–6 ppt in air [79]	Leucine [73]	
	2-Methylbutanal	Malty/Chocolate	1 ppb in water [83]	Isoleucine [73]	
	Methional	Potato	0.1–0.2 ppt in air [78]	Methionine [74]	
UHT milk	Hydrogen sulfide	Rotten eggs	10 ppb in water [85]	Thiamine or cysteine [48]	[48,91]
	Methanethiol	Rotten cabbage	0.2 ppb in water [92]	Methionine [74]	
	2-Methylpropanal	Pungent	1 ppm in water [93]	Valine [73]	
	2-Furaldehyde	Woody	3 ppm in water [62]	Sugar [94,95]	
